# SCHISTOACT: a protocol for an open-label, five-arm, non-inferiority, individually randomized controlled trial of the efficacy and safety of praziquantel plus artemisinin-based combinations in the treatment of *Schistosoma mansoni* infection

**DOI:** 10.1186/s13063-023-07790-3

**Published:** 2023-11-27

**Authors:** Charles O. Obonyo, Vincent O. Were, Peter Wamae, Erick M. O. Muok

**Affiliations:** Centre for Global Health Research, Kenya Medical Research Institute, P.O. Box 1578-40100, Kisumu, Kenya

**Keywords:** Schistosomiasis, Artemisinin, Praziquantel, Combinations, Children, Randomized controlled trial

## Abstract

**Background:**

Schistosomiasis control relies on praziquantel for preventive chemotherapy. Alternative drugs are needed for the treatment and control of schistosomiasis. Praziquantel is effective against adult schistosome worms but ineffective against larval stages of the parasite and cannot prevent re-infection or interrupt the transmission of infection. Continued reliance on praziquantel for wide-scale schistosomiasis control will likely accelerate the emergence of drug resistance. Artemisinin derivatives are effective against the juvenile stages but ineffective against adult worms. The SCHISTOACT study aimed to evaluate the efficacy and safety of praziquantel plus one of four artemisinin-based combinations in treating *Schistosoma mansoni* infection in Kenya.

**Methods:**

The SCHISTOACT study is an open-label, head-to-head, five-arm, proof-of-concept, non-inferiority, individually randomized controlled trial with a follow-up of 12 weeks. A total of 540 primary school-aged children from the Mwea area, Kirinyaga County in central Kenya, diagnosed with *S. mansoni* infection (by Kato-Katz method) are randomly allocated (1:1:1:1:1) to a single dose of praziquantel plus a 3-day course of artesunate-sulfalene/pyrimethamine, or artesunate-amodiaquine, or artesunate plus mefloquine, or dihydroartemisinin-piperaquine, or praziquantel control arm. The primary endpoints are efficacy (cure rate, assessed by microscopy) and safety (adverse events) of each study arm 6 weeks after treatment. Secondary endpoints include cumulative cure rate, egg reduction rate, and re-infection 12 weeks after treatment. The non-inferiority margin is set at − 10 for the risk difference in cure rates between praziquantel and the combined treatment.

**Discussion:**

This study assesses a strategy for repurposing artemisinin-based combination therapies (ACTs) for treating schistosomiasis. It adopts a head-to-head comparison of four different ACTs to test a non-inferiority hypothesis and to strengthen local capacity to conduct clinical trials for interventions against neglected tropical diseases.

**Trial registration:**

Pan-African Clinical Trials Registry PACTR202001919442161. Retrospectively registered on 6 January 2020

**Supplementary Information:**

The online version contains supplementary material available at 10.1186/s13063-023-07790-3.

## Introduction

Human schistosomiasis is an important but neglected debilitating water-borne parasitic disease caused by trematode worms of the genus *Schistosoma*. The three most common schistosome species that infect man include *Schistosoma mansoni*, *S. haematobium*, and *S. japonicum* [1]. Human schistosomiasis presents clinically as an intestinal or urogenital form. The global burden of schistosomiasis is estimated at 1.4 to 3.3 million disability-adjusted life years annually [2]. Over 90% of the 250 million people infected with schistosomiasis reside in sub-Saharan Africa, where *S. mansoni* and *S. haematobium* are the most prevalent [3]. The high-risk groups for schistosomiasis include preschool-aged and school-aged children and adults with occupations that involve contact with infested water, such as fishermen, car washers, irrigation workers, farmers, and women doing domestic chores [4].

The global schistosomiasis control strategy recommended by the World Health Organization (WHO) is based on preventive chemotherapy [5]. Praziquantel is currently the only drug available for preventive chemotherapy; it is effective against all the species of human schistosomiasis; it can be administered as a single oral dose, is affordable, safe, and partially effective [6]. Praziquantel is effective against the invasive stages and adult schistosome worms but less effective against the parasite’s juvenile stages (schistosomula) [7]. Partly for this reason, praziquantel is not completely curative and does not prevent re-infection [8]. The reliance on a single drug for wide-scale community preventive treatment exerts a high drug pressure and could favor the emergence of praziquantel-resistant parasites. Evidence from laboratory studies suggests reduced susceptibility of schistosomes to praziquantel [9, 10]. Despite these limitations, mass administration of praziquantel has substantially reduced schistosomiasis-associated morbidity and mortality [11]. Several strategies are proposed to preserve the potency of praziquantel, including increased doses [12], repeated doses [13], or increased frequency of administration [14, 15]. The drug development pipeline for schistosomiasis is empty, and there is an urgent need to evaluate new drugs to replace or complement the use of praziquantel. In these circumstances, drug repurposing is an optimal approach to overcome the obstacles of cost and time needed to develop new drugs by exploring new indications for approved medicines currently used to treat other conditions [16]. Combination therapy is a promising strategy to improve praziquantel efficacy and delay the development of drug resistance. Possible candidates for combination therapy include praziquantel plus antimalarials such as mefloquine or artemisinin derivatives [17].

The artemisinin derivatives (artesunate, artemether, and dihydroartemisinin) are promising candidates for the treatment and chemoprophylaxis of schistosomiasis [18, 19]. This class of drugs is currently the most potent for treating malaria, for which they are used as artemisinin-based combination therapy (ACT) [20]. Artemisinin derivatives are specifically effective against the juvenile worms of the parasite [17, 19]. These differences in the mechanism of drug action support a theoretical basis for combining praziquantel with an ACT in treating schistosomiasis. The combination is likely synergistic, acting at two stages of the schistosome life cycle to cure the primary infection and block further transmission, especially in high-transmission areas. Data on the efficacy and safety of ACTs in treating schistosomiasis are scarce. However, compared to praziquantel, ACTs demonstrated significantly lower cure rates in treating children with schistosomiasis [21–24]. The results of the two studies that compared the efficacy of praziquantel plus dihydroartemisinin-piperaquine or praziquantel plus artesunate-mefloquine to praziquantel alone in the treatment of children with schistosomiasis were inconsistent [25, 26]. In one small study, praziquantel plus artesunate-mefloquine showed no benefit in the treatment of *S. haematobium*, while in a large trial, praziquantel plus dihydroartemisinin-piperaquine was associated with a substantial benefit in the treatment of *S. mansoni* [25, 26]. Hence, the additional benefit of adding an ACT to the standard praziquantel treatment for schistosomiasis remains unclear.

There is no systematic data from a single study comparing the efficacy of praziquantel plus an ACT to praziquantel alone on schistosomiasis. However, a systematic review of the available scanty evidence concluded that combining an artemisinin derivative plus praziquantel was more effective than praziquantel monotherapy [27]. WHO has approved at least five ACTs for the treatment of uncomplicated malaria: artemether-lumefantrine, artesunate plus amodiaquine, artesunate plus sulfadoxine-pyrimethamine, artesunate plus mefloquine, and dihydroartemisinin-piperaquine [20]. It is unclear which ACTs are effective and safe for treating schistosomiasis when combined with praziquantel.

## Methods

### Study objectives

The overall purpose of the SCHISTOACT study is to evaluate the role of combination therapy in treating schistosomiasis. The primary objective of the study is to evaluate the efficacy (assessed as cure rate by microscopy) and safety (frequency of adverse events) of a single dose of praziquantel combined with a 3-day course of artesunate plus sulfalene/pyrimethamine or artesunate plus amodiaquine or artesunate plus mefloquine or dihydroartemisinin-piperaquine compared with single-dose praziquantel in the treatment of children with confirmed *Schistosoma mansoni* infection.

Our secondary objectives are as follows:

- To assess the cumulative cure rate at 12 weeks after treatment
- To assess the egg reduction rate at 6 and 12 weeks after treatment
- To assess the re-infection rate at 12 weeks after treatment

### Study design

**Fig. 1:**
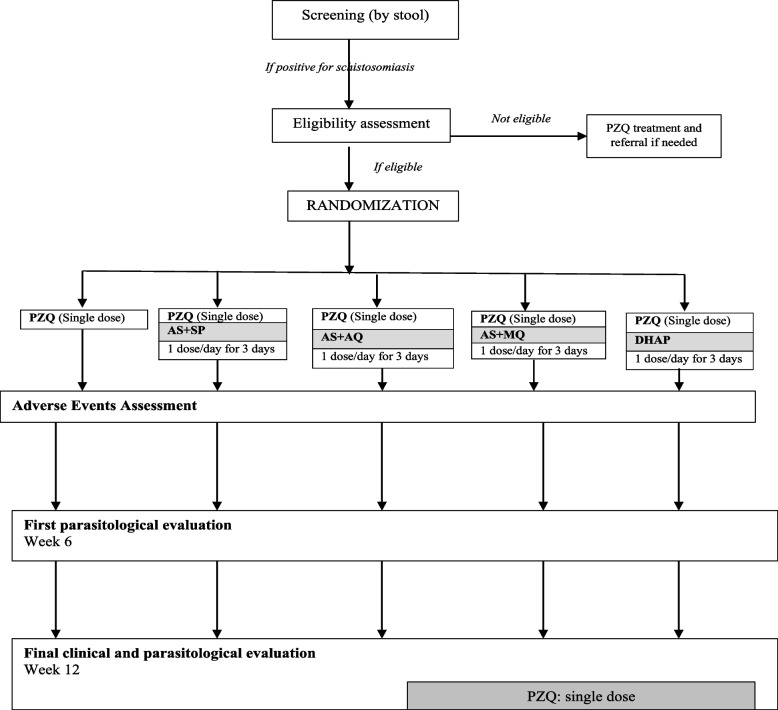
Study flow chart

The SCHISTOACT study is designed as a phase III, open-label, five-arm, head-to-head, proof-of-concept, individually randomized, controlled, non-inferiority trial. The participants are randomly allocated (ratio 1:1:1:1:1) individually to one of five treatment groups. At baseline, participants in the control arm receive praziquantel (PZQ, single dose of 40 mg/kg). Those in the intervention arms receive a single dose of praziquantel (40 mg/kg) plus a 3-day course of one of four artemisinin-based combination therapies (4 mg/kg of the artemisinin component, once a day for three consecutive days, as fixed-dose WHO-recommended combination treatment for uncomplicated falciparum malaria). The four artemisinin-based combination therapies (ACTs) that are combined with praziquantel are artesunate plus sulfalene/pyrimethamine, artesunate plus amodiaquine, artesunate plus mefloquine, and dihydroartemisinin-piperaquine. These four ACTs were selected because they are widely available for malaria treatment, co-formulated, for once-daily dosing therapy, have a well-established safety profile and have all been evaluated for treating schistosomiasis with inconclusive results. The 6-week interval between the administration of study treatment and evaluation of primary endpoints has been chosen because it corresponds to the duration of schistosome larval maturation. The participants will be followed up at 6 and 12 weeks. During these follow-up visits, stool samples will be collected, and clinical assessments will be performed. All children will receive a standard dose of praziquantel at the end of the study. The study flow chart is shown in Fig. 1.

### Study area and population

This study population comprises primary school-age children (9–15 years) from selected primary schools in the Mwea East and Mwea West sub-counties in Kirinyaga County, central Kenya. The schools are eligible for inclusion if they have at least 50 pupils infected with *S. mansoni* detected from a screening exercise*.* The area is endemic for *S. mansoni*, with up to 75% prevalence in school children [28]. The major socio-economic activities in the study area are rice growing under irrigation and small-scale subsistence farming. Malaria transmission in the area is extremely low (< 1/1000 individuals), and elimination is envisaged.

### Sample size

The sample size is calculated using the statistical test for proportions based on the hypothesis that combination therapy using praziquantel plus an artemisinin-based combination is not inferior to praziquantel alone in treating schistosomiasis. Assuming a cure rate of 72% with praziquantel based on longitudinal studies from western Kenya [29], a cure rate of 62% (maximum difference of 10%) is considered clinically acceptable for non-inferiority of praziquantel plus an ACT combination therapy. With this non-inferiority margin, a power of 80%, and a one-sided alpha value of 0.025, a sample size of 102 children is needed in each treatment arm. To account for a 5% loss to follow-up, the total number of children to be enrolled and randomized is 540 (108 in each study arm).

### Eligibility criteria

Children are eligible for the trial if they meet the following inclusion criteria:

- Age between 9 and 15 years old (confirmed from the date of birth recorded on the school registers)
- Enrolled in a participating primary school in the Mwea sub-county, Kirinyaga County
- Confirmed *S. mansoni* infection (confirmed by eggs in stool)
- A resident of Mwea sub-county, Kirinyaga County, central Kenya
- Able to take oral treatment
- Signed informed consent by parent/guardian and oral assent from the child

Children who meet any of the following criteria will be excluded from participation in the study:

- Bodyweight ≥ 50 kg
- Hemoglobin level ≤ 8.0 g/dL
- Co-infection with *Plasmodium falciparum* infection
- Signs of severe malnutrition (evidenced by severe wasting [defined as weight-for-height < − 3SD] or mid-upper arm circumference [MUAC] < 12 cm)
- Current or history of convulsions
- History of hypersensitivity to artesunates, sulfonamides, amodiaquine, mefloquine, or praziquantel
- History of treatment using an anti-malaria or anti-schistosomal drug within 28 days before enrollment in the study

### Randomization and blinding

The randomization (in blocks of 15) list was computer-generated by an independent statistician not involved in participant management. The resulting sequence (randomization number and treatment arm) is stored in opaque sealed envelopes. The enrolling clinician assigns a study number to the participant. The study nurse implements the treatment allocation by opening a sealed envelope for each child according to the assigned randomization number. The laboratory staff are blinded to the treatment allocation, but participants, clinical staff, and data analysts are not.

### Study procedures

#### Screening, enrollment, and participant follow-up

Potential participants were approached through the primary school system. At screening, every child provides a fresh stool (about 5 g) sample, which is used to detect the presence of *S. mansoni*. The geohelminths (hookworms, *Ascaris lumbricoides*, and *Trichuris trichiura*) are recorded. A capillary blood sample is taken by fingerprick to estimate hemoglobin and determine the presence of malaria parasites. Every child who tests positive for *S. mansoni* is invited for confirmation of eligibility. Eligible children will be recruited until the sample size is attained. The benefits of treatment for schistosomiasis and the availability of free medical check-ups were explained to potential participants to encourage participation and to ensure that adequate numbers were enrolled. Ineligible children are treated using a single oral dose of PZQ and referred for further treatment if need be.

At enrollment, verbal assent from the child and written informed consent from the parent/guardian are obtained. The study clinician takes a standard baseline medical history and performs a clinical examination, including weight (using a digital weighing scale), height (using a stadiometer), and temperature (using a digital thermometer) measurements. The clinician also assesses the size of the liver and spleen. All this information will be recorded on a paper-based case record form, which the principal investigator reviews to ensure compliance with the study protocol. Children receive study treatment according to the randomization list. Administration of all the study drugs is directly observed by the study nurse. All study drugs are given orally by the study nurse. For each child, the nurse records whether they have received their scheduled treatment, the dose, and reasons for missing treatment. A copy of the treatment sheet with dosing regimens is provided as supplemental material (Additional file 1: Appendix 4).

Children are followed up at weeks 6 and 12 after starting treatment. During the first 3 days after enrollment, they are visited at school or home daily to administer study treatment and complete the adverse events questionnaire. At the week 6 and 12 follow-up visit, the children provide an early morning stool sample, and the study clinician takes a brief medical history, performs a clinical examination, and obtains a fingerprick blood sample for hemoglobin estimation. Participants who do not return for the scheduled follow-up visits are visited at home.

A detailed schedule of the study procedures at different time points is presented in Table 1.

**Table 1:** Scheduled visits and procedures

	Enrollment/baseline	Allocation	Intervention (3 days)			Follow-up	
	− D5	D0	D0	D1	D2	Week 6	Week 12
**Enrollment**							
Stool examination	X						
Eligibility screen	X						
Informed consent/assent		X					
Child assent		X					
Randomization		X					
**Intervention**							
Praziquantel alone			X				
Praziquantel plus ACT^a^			X	X	X		
**Assessment**							
Baseline assessment							
Body weight and height	X					X	X
Hemoglobin (HemoCue)	X					X	X
Outcome assessment							
Stool examination						X	X
Safety assessment							
Adverse events	X		X	X	X	X	

^a^ACT includes any of artesunate plus sulfalene-pyrimethamine, artesunate plus amodiaquine, artesunate plus mefloquine, or dihydroartemisinin-piperaquine

#### Parasitological monitoring of response to treatment

All the laboratory tests are performed by the laboratory at the sub-county hospital, using standard techniques. To assess for the presence of *S. mansoni*, participants provided a fresh stool sample. Duplicate slides are prepared from the stool sample and examined under the microscope independently by two experienced laboratory technicians. The *S. mansoni* and soil-transmitted helminth eggs are quantified by use of the Kato-Katz fecal thick-smear technique, with a template containing about 41.7 mg of feces when filled. The number of *S. mansoni* eggs is counted per slide, and the mean of the two readings is multiplied by 24 to express them as eggs per gram (EPG) of feces. The intensity of infection is categorized according to the WHO classification as light (1–99 EPG), moderate (100–399 EPG), or heavy (≥ 400 EPG) [30]. As a quality control measure for interobserver variability, a third technician reads a random selection of 10% of slides, and all slides for which the initial readings vary by more than 20% between the two technicians.

### Hemoglobin assessment

The study clinician takes a fingerprick blood sample at screening and week 6 and week 12 follow-up visits to determine the hemoglobin level. The hemoglobin level is estimated using a portable hemoglobinometer (HemoCue Hb 301, HemoCue, Angelholm, Sweden). The hemoglobin value obtained is categorized according to the WHO criteria [31].

### Treatment

The participants are allocated individually to treatment groups. The treatments administered in this study are as follows:

- Praziquantel (PZQ) (Biltricide, Bayer Healthcare, Leverkusen, Germany) is a tablet with 600 mg active ingredient. PZQ is administered as a single oral dose of 40 mg/kg body weight.
- Artesunate plus sulfamethoxypyrazine/pyrimethamine (AS+SP) (Coarinate Junior, Dafra Pharma, Turnhout, Belgium) is a fixed-dose combination tablet consisting of 100 mg artesunate, 250 mg sulfamethoxypyrazine, and 12.5 mg pyrimethamine. Participants receive a dose of artesunate plus sulfalene/pyrimethamine (4 mg/kg of the artesunate component) tablets once daily for 3 days.
- Artesunate plus amodiaquine (AS+AQ) (ASAQ, Winthrop, Sanofi-Aventis, France) is a fixed-dose combination tablet consisting of 100 mg artesunate and 270 mg amodiaquine. Participants receive a dose of artesunate plus amodiaquine (4 mg/kg of the artesunate component) tablets once daily for 3 days.
- Artesunate plus mefloquine (AS+MQ) (ARTEQUIN, Acino/Mepha, Switzerland) is a fixed-dose combination tablet consisting of 200 mg artesunate and 250 mg mefloquine. Participants receive a dose of artesunate plus mefloquine (4 mg/kg of the artesunate and 8.3 mg/kg/day of mefloquine) tablets once daily for 3 days.
- Dihydroartemisinin-piperaquine (DHAP) (D-ARTEPP, Guilin Pharmaceutical Co. Ltd, China) is a fixed-dose combination tablet containing 40 mg of dihydroartemisinin and 320 mg of piperaquine. Participants receive a dose of dihydroartemisinin-piperaquine (4 mg/kg/day of dihydroartemisinin and 20 mg/kg/day of piperaquine) tablets once daily for 3 days.

### Criteria for withdrawal or discontinuation from the study

The objective criteria for discontinuation from study medication and/or institution of another form of treatment include the following:

- Parent/guardian’s withdrawal of consent
- Development of a concomitant illness that would interfere with the clear interpretation of study outcomes
- Inability to retain study medication due to vomiting
- Any serious adverse event requiring treatment withdrawal in the opinion of the study clinician or at the request of a parent/guardian
- Major protocol violation, omission of more than one treatment dose, or a credible report of self-medication with an additional anti-schistosomal or antimalarial drug outside the study protocol

### Adverse events

The study nurse administers all the study drugs. Children will ingest the study drugs with a small amount of water. Before administering the study treatment, all the children are given a light meal (slices of bread and a glass of orange juice) to reduce the study drugs’ nauseating effect and improve drug bioavailability [32]. Participants are observed for 1 h after drug ingestion to ensure retention and to assess for any immediate adverse events. Participants who vomit within 1 h of drug ingestion are re-dosed. Participants with repeated vomiting are withdrawn from the study.

All adverse events are elicited by asking the children about any adverse drug reactions observed after taking the study drugs. The symptoms will be assessed, graded (according to their intensity as perceived by the child) by the study clinician, and recorded on the adverse event form. The adverse events form is administered an hour after study treatment and 24 h later (before the next ACT dose). The following information is recorded on the adverse events form: adverse event description, duration (start and end dates); severity grade (mild, moderate, severe); causality (relationship to the study drugs); action(s) taken; and the outcome. The participants who develop adverse events (AEs) or serious adverse events (SAEs) are treated appropriately and followed up until they return to a normal state of health. The investigator reports any SAE occurring or observed during the follow-up period to the KEMRI Scientific and Ethics Review Unit within 48 h of being aware of the event.

### Concomitant treatment

According to the national treatment guidelines, all children with helminthic infections are treated with a single dose of 400 mg of albendazole. A list of prohibited or contra-indicated medicines was availed to the study clinicians. Participants are prohibited from taking any non-study drugs during the study. The study clinician evaluated any reports of non-study medications taken to assess for possible drug interactions.

### Outcomes

The primary efficacy outcomes are as follows:

- Cure rate at week 6 after starting study treatments, as assessed by microscopy. The cure rate on each PZQ plus ACT arm is compared with that on the PZQ alone arm.
- The number of participants with drug-related adverse events in the first 24 h after study treatment.

The secondary outcomes are as follows:

- Cumulative cure rate at week 12 after enrollment, as assessed by microscopy. The cure rate on each PZQ plus ACT arm is compared with that on the PZQ alone arm.
- Egg reduction rate at weeks 6 and 12 after starting treatment, as assessed by microscopy and intensity reduction rate, comparing each PZQ plus ACT with PZQ alone arm.
- The prevalence of re-infection at 12 after starting treatment, as assessed as the number of children who were cured at week 6 follow-up but positive for *S. mansoni* at the week 12 follow-up, compared each PZQ plus ACT arms to the PZQ alone arm.

### Data management and analysis

Data collected from each participant is recorded on paper-based case report forms (CRFs) in black ballpoint pen. The principal investigator will ensure that the data in the CRFs are accurate, complete, and legible. Data from the case record forms is entered by a data clerk into computers as they accrue using the MS Access program. The data is verified by comparing it with the CRFs. Missing data is imputed as either treatment success or treatment failure (assuming the same infection intensities before and after treatment). Validated data is transferred to SPSS version 20 and STATA version 12 for statistical analyses. The CRFs and the electronic data will be kept for 5 years after the primary manuscripts are published.

Data is analyzed using SPSS for Windows version 20 (SPSS Inc., Chicago, IL) and STATA version 12.0 (StataCorp., College Station, TX, USA) packages. A two-sided *p*-value less than 0.05 is considered statistically significant for all tests.

Demographic and other baseline characteristics are compared to check for imbalances between the study groups. These measures will be tabulated from the intent-to-treat population. Significance tests are not performed to compare the differences between study groups at baseline. Where imbalances are suspected, additional analyses are performed to adjust for these variables.

To determine efficacy, both the per-protocol and intention-to-treat analyses are done, but the per-protocol analysis is the main approach. The primary efficacy outcome is the cure rate, defined as the proportion of children infected with *S. mansoni* at enrollment but not excreting eggs by week 6 after taking the study treatment. In the computation of the cure rate, children randomized to the PZQ alone study arm are considered the control group. Cure rates are compared between the treatment groups using Pearson’s chi-square test for contingency tables (in a per-protocol analysis) and summarized as absolute risk differences with 95% confidence intervals (CI). The per-protocol population is defined to include all participants who were randomized and who completed the study procedures to week 6 or 12. The primary hypothesis for non-inferiority is assessed by considering the lower limit of the 95% CI for the risk difference. Non-inferiority is concluded if the lower bound of the 95%CI from the risk difference does not exceed the non-inferiority margin of − 10.

The cumulative cure rate at week 12 is assessed by calculating a two-sided difference in proportions of cured children from each PZQ plus ACT arm compared with the PZQ alone arm. Arithmetic mean (AM) egg counts are computed for all non-cured participants. The egg reduction rate is computed as (1 − (AM EPG after treatment/AM EPG before treatment)) × 100. The ERR is expressed as a percentage. Re-infection rate is the proportion of children who test negative for *S. mansoni* at week 6 but test positive at week 12 follow-up. Changes in continuous variables over time are assessed using analysis of variance (ANOVA). The least significant difference will be used to assess post hoc pair-wise comparisons between the group means. Logistic regression will be used for analyses adjusted for the treatment received.

For the primary safety analysis, the frequency and pattern of adverse events are computed by patient counts and percentages in each of the five treatment groups. Adverse events are computed for all participants who received at least one dose of the study drug (intention-to-treat population).

### Ethics and dissemination

#### Ethics approvals

The research protocol (version 1.2 dated January 2018) for this study has been approved by the Scientific and Ethics Review Unit (SERU) at Kenya Medical Research Institute (KEMRI), Nairobi (on 12 March 2018, Ref. SERU# 3507). The trial is registered with the Pan-African Clinical Trials Registry, PACTR202001919442161. The trial is conducted in compliance with the Declaration of Helsinki and the ICH Good Clinical Practice (GCP) guidelines. Approvals for changes to the protocol (for example, amendments) will be sought from the ethics committee, and the approved changes communicated to the study team and participants.

#### Study and data monitoring

The principal investigator and the study investigators oversee the implementation of the study, including adherence to the protocol, participant recruitment, retention and follow-up, administration of the study drugs and compliance monitoring, and quality of the accruing data. Partly due to the established safety profile of the study drugs, and the duration of the study we have not established a data safety monitoring board and interim analysis is not planned.

#### Consenting process

A meeting was held with parents, teachers, and community leaders to inform them about the study’s aims, procedures, potential benefits, and risks. Permission to conduct the study is obtained from the Kirinyaga County Commissioner, the Kirinyaga County Directorates of Health and Education, and the respective school administration. School children in grades 3 to 5, aged between 9 and 15 years old, are informed about the study and asked to provide a stool sample to be tested for *S. mansoni*. Children and parents/guardians of all children who test positive for *S. mansoni* are invited to a meeting where general information about schistosomiasis, the aims of the study, procedures, potential risks, and benefits are explained in the local languages (Swahili and Kikuyu) and in a culturally sensitive way. The study nurse obtains written informed consent from the parents/guardians and verbal assent from the child. All the study personnel are trained in GCP. We explain that no biological samples are stored for any ancillary studies. A copy of the child assent and parental informed consent used in the study is provided in an online supplemental file (Additional file 1: Appendices 1–3).

#### Withdrawal

Participation is voluntary, and participants can withdraw from the study at any time and for any reason without consequences. Participants will be withdrawn from the study due to repeated vomiting or occurrence of SAE. Children with SAEs are followed up until the end of 6 weeks after the last treatment dose. Participants withdrawn from the study due to medical reasons are referred to adequate health facilities for further care. Unless consent is withdrawn, efforts are made to assess these children at the week 6 follow-up schedule.

Valid reasons for study discontinuation include completion of the scheduled follow-up period and exclusion from follow-up. Randomized participants who miss one or more scheduled follow-up visits remain in the study and are followed up as planned. Loss to follow-up is expected to be minimal because all the participant-related study procedures are completed at school and, if necessary, at home. In the unlikely event that a study participant moves away from the study area during the study period, they are considered lost to follow-up and are not included in the efficacy analysis. However, data collected up to the time of withdrawal will remain in the study and be used for analysis of whether or not the participant continues with the follow-up visits. Participants who have been withdrawn from the study are administered a standard dose of praziquantel after the final study visit. No additional participants are recruited to replace those who have withdrawn.

#### Risks, benefits, and compensation

All potential participants will be informed that they will not receive any cash payments for participating in the study. However, the project will cover the costs of referral and hospitalization in case of serious adverse events. All participants will receive free and close medical follow-up while in the study.

#### Confidentiality

Personal information generated during the study is kept confidential according to the Kenyan Data Protection Regulations, 2021 requirements. Access to all paper and electronic files is restricted to authorized study staff. In rare circumstances, this information may be made accessible to the ethics committee and the research regulatory authorities. All the personal information collected during the study is stored in a database using unique personal identification codes.

#### Dissemination

The reporting of this study protocol is based on the Standard Protocol Items Recommendations for Intervention Trials (SPIRIT) recommendations. The SPIRIT checklist is attached. The results of this study will be disseminated regardless of the outcome. The findings will be shared with local and national policymakers, presented at relevant national and international scientific conferences, and published in peer-reviewed journals according to the Consolidated Standards of Reporting Trials (CONSORT) guidelines. All the investigators will participate in the authorship of the manuscripts from the study. Professional medical writers will not be engaged. The participant-level data generated during the study will be available to other researchers through a data-sharing agreement.

### Trial status

The current protocol version is 1.2 approved on 12 March 2018. The recruitment of children started on 26 September 2022 and was finalized on 30 November 2022. The trial is currently in the phase of final follow-up. The follow-up will continue until September 2023. The trial start-up was delayed by funding challenges and a national election.

## Discussion

THE SCHISTOACT is the first clinical trial to compare the efficacy and safety of praziquantel plus four artemisinin-based combinations with praziquantel alone in treating African children with intestinal schistosomiasis. The role of artemisinin-based combination therapies in schistosomiasis control cannot be deduced from the available scanty evidence. Due to the differences in the mechanism of drug action, combination therapy using praziquantel plus an ACT has the potential to support the move from morbidity to transmission control, thus accelerating the global schistosomiasis elimination agenda. If the effectiveness and safety are confirmed, combination therapy is likely to be useful for schistosomiasis control in malaria-free settings, in patients with both malaria and schistosomiasis, as chemoprophylaxis for travelers to schistosomiasis-endemic areas, and as second-line treatment in cases of praziquantel failure. The strength of this study lies in the head-to-head comparison to contribute comprehensive evidence from a single study regarding the comparative efficacy and safety of praziquantel combined with four different artemisinin-based combination therapies in the treatment of *S. mansoni*. Overall, this study will contribute much-needed evidence to inform the global policy on the role of artemisinin-based combination therapies in schistosomiasis control.

### Supplementary Information

**Additional file 1: Appendix 1.** Assent form. **Appendix 2. **Informed consent explanation. **Appendix 3. **Informed consent agreement/certificate. **Appendix 4. **Treatment dosing table.

**Additional file 2.** SPIRIT checklist.

## Data Availability

Following completion of the study, project data will initially be used by the study team to prepare manuscripts. The final trial data will be available for consultation and publically available via direct requests to the principal investigator.
