# Supplementary material for: SCHISTOACT: a protocol for an open-label, five-arm, non-inferiority, individually randomized controlled trial of the efficacy and safety of praziquantel plus artemisinin-based combinations in the treatment of Schistosoma mansoni infection
Source: Trials. 2023 Nov 27;24:763. doi: 10.1186/s13063-023-07790-3 (PMC10683197; doi:10.1186/s13063-023-07790-3)
Supplement: Supplementary file 1 — Additional file 1: Appendix 1. Assent form. Appendix 2. Informed consent explanation. Appendix 3. Informed consent agreement/certificate. Appendix 4. Treatment dosing table. [file 13063_2023_7790_MOESM1_ESM.docx]

# APPENDIX 1: Assent Form

ASSENT EXPLANATION FORM FOR CHILDREN PARTICIPATING IN THE BILHARZIA TREATMENT STUDY

**
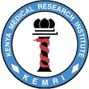
**

**TITLE OF STUDY: Efficacy and safety of Praziquantel alone or in combination with Artemisinin-based combinations in the treatment of children with** *Schistosoma mansoni* **in Mwea, Kirinyaga County.**

**INVESTIGATORS AFFILIATION**

PROF. CHARLES O. OBONYO KEMRI

MR. VINCENT WERE KEMRI

MR. PETER WAMAE KEMRI

Dr ERICK MUOK KEMRI

PROF. SAMMY NJENGA KEMRI

**Introduction**

We are researchers from the Kenya Medical Research Institute (KEMRI). We are conducting a study in your school to find out the best treatment for school children with a disease called Bilharzia. We are asking you to take part in this study. Bilharzia is a very common disease in this area, especially among children who have regular contact with contaminated water. A person with Bilharzia can either pass red-coloured urine or complain of abdominal pains and blood-stained stool. In school children, it may lead to poor academic performance as well as stunted growth. Bilharzia can stay in the body for a very long time because it doesn’t cause people to be seriously sick. The medicine for treating bilharzia is called Praziquantel, but research has shown that it is becoming weak and not curing all people with bilharzia. In our research, we want to test other medicines for treating people with bilharzia.

**WHO WILL BE IN THIS STUDY:**

We will ask school children who are between the ages of 6 and 15 years to take part in the study if we have found eggs of Bilharzia in their stools, they normally live in the Mwea area and have met the other requirements for taking part in this research.

**WHAT WILL HAPPEN IN THE STUDY?**

If you agree to take part in this study, we will ask you some questions related to your health, we will also ask you to provide a sample of stool for testing to confirm if you have bilharzia. Only those children whom we find eggs of bilharzia in their stool will be asked to take part in the study. All the children who enter the study will be divided into five different groups and each group will be treated using a different combination of medicines. We have five different medicines that we are using for treating people with bilharzia. The treatment will be given only once or once every day for three days by our study nurse. Every day before the nurse gives medicine to the children, she/he will ask if anybody vomited or became sick after yesterday’s medicine. We will visit your school again after one month and 3 months. When we visit, we will ask you for a stool sample to test to confirm that you are cured of bilharzia. Today and next time we visit your school, we will ask you to take a small sample of blood from your fingers to test if you have malaria and to measure the level of blood in the body. We will not take any stool or blood samples to store for additional tests.

**WHOM CAN YOU CONTACT IF YOU HAVE A QUESTION?**

If you have any questions about taking part in the study, please call Prof Charles Obonyo [Tel 0724993118] and feel free to ask any questions or you can request your parent/guardian to ask the questions on your behalf. If you have any questions about your rights as a study participant, please contact the Scientific and Ethics Research Unit (SERU) at KEMRI, Nairobi, through Telephone no. 0717719477. We will give you a signed copy of this form to take home.

**AGREEMENT TO TAKE PART IN THE STUDY**

**Your signature below indicates that:**

1. You have had an opportunity to discuss this study and ask questions and you are satisfied with the answers.
2. You agree to participate in this study.
3. You understand that nobody is forced to take part in this study, and even after agreeing to take part you can still ask to stop taking part
4. You understand that you can only take part in the study if your parent/guardian also agrees.

_______________________

Name of study participant (please print)

_______________________

Signature of participant Date

_________________

Thumb-print of participant Date

________________________

Name of Witness (please print)

___________

Signature of Witness Date

_____________________________________________________________________________

Name of the person obtaining assent

____________

Signature of Person Obtaining Assent Date

#

# APPENDIX 2: INFORMED CONSENT EXPLANATION

INFORMED CONSENT FORM FOR PARENTS OF CHILDREN PARTICIPATING IN THE BILHARZIA TREATMENT STUDY IN KIRINYAGA COUNTY


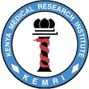


**TITLE OF STUDY: Efficacy and safety of Praziquantel alone or combined with Artesunate-based combinations in the treatment of children with *Schistosoma mansoni* in Mwea, Kirinyaga County**

**PRINCIPAL INVESTIGATOR**: PROF. CHARLES O. OBONYO

**CO-INVESTIGATORS:**

MR. PETER WAMAE

MR. VINCENT WERE

Dr. ERICK MUOK

PROF. SAMMY NJENGA

**INSTITUTION:**  KENYA MEDICAL RESEARCH INSTITUTE (KEMRI)

**PARTICIPANT INFORMATION SHEET (Flesch-Kincaid reading score=7.0)**

We, the Kenya Medical Research Institute (KEMRI) are doing a research study to evaluate the best treatment for bilharzia in school children. We invite you to allow your child to take part in this study. Before you provide permission for your child to take part or not, you need to understand why the research is being done and what it will involve. Please take time to read the following information carefully. Ask us if there is anything that is not clear or if you would like more information. Take time to decide whether you wish to have your child join the study.

**What is the purpose of this study?**

Bilharzia is a common disease in this area, especially among children and those who stay close to water masses. Bilharzia is acquired by regular contact with contaminated water. There are two main types of Bilharzia: in one, the patient passes blood-stained urine and in the other, the disease affects the intestines causing abdominal pains and the patient passes blood-stained stool. In this study, we will focus on the treatment of both types of bilharzias. The disease can stay in the body for a long time and in school, children may lead to frequent tiredness, poor academic performance, reduced levels of blood as well as stunted growth. The medicine for treating people with bilharzias is called Praziquantel. The result of recent research done in our country and elsewhere shows that Praziquantel is losing its strength in curing people with bilharzia. This means that in the future new stronger medicines must be discovered for treating bilharzia. Our research aims to find the best treatment for treating children with bilharzia. To do this, we will compare the ability of Praziquantel when used alone or when combined with medicines currently being used for malaria in curing children with bilharzia.

**Why has your child been chosen?**

Bilharzia is a common disease among children who play in contaminated water. We will therefore ask the permission from parents of 540 children who reside and attend school in the Mwea area of Kirinyaga County. We have selected a few schools in which to do this research and your child’s school is one of them.

**Does my child have to take part?**

It is up to you to decide if you want your child to take part in the research or not. Participation in this research is purely voluntary. If you decide that your child takes part, you will be asked to sign a parental permission form. If you permit your child to take part, he/she is still free to withdraw at any time without giving a reason. A decision to withdraw at any time or a decision to not take part, will not affect the standard of care your child will receive.

**What will happen to my child if I permit him/her to take part?**

*Enrollment procedures*

Your child has been found to have a disease called bilharzia. If you agree for your child to take part in this study, we will ask him/her some questions about his/her health, examine him/her, and then prick his/her finger to take a small amount of blood (1/4 of a teaspoon). We will use the blood to test for malaria germs and count the blood cells. These blood tests will be done in the laboratory at Kimbimbi sub-County Hospital. We will not take any stool or blood samples to store for additional tests.

*When does the study treatment start?*

If you permit your child to join, the study starts two days from now. Between today and then, we will examine your child’s stool and blood to confirm that he/she can participate in the study.

*How will they decide what treatment my child will get?*

Because we do not know the best way to treat children with bilharzia, we will make comparisons. All the children will be divided into 5 groups. Each group will get a different treatment and the outcomes of these treatments will be compared. The groups are selected by a computer, which has no information about the individual. In other words, the choice of treatment group for your child will be by chance. Your child has a chance of 1 in 5 being in one of the treatment groups. You may not choose or change which group your child will be in.

*Who will give the treatment?*

Our study nurse will give the study medicine to your child at the school. All the children in each of the four groups will receive treatment as crushed tablets with water. Your child will receive the medicines only once per day.

*What will happen for the rest of the time?*

After treatment, we will visit your child at school after six weeks and thereafter, after 3 months after entering the study. During these visits, we will evaluate your child to see how well the medicines have worked. At each of the follow-up visits, we will ask him/her to provide a stool sample, ask him/her questions about his/her health, measure his/her temperature, and examine and weigh him/her. At the week 6 visit, we will take a few drops of blood from your child by pricking his/her finger (less than 1/4 of a teaspoon). The blood will be used to test for the level of his/her blood and the presence of malaria. We will provide treatment if your child is ill at any of the visits.

*How much time does it take?*

Each follow-up visit will take less than 30 minutes. The total time that each child takes part in the study is 3 months. In total, the study will involve 540 schoolchildren.

**What do I have to do?**

If your child joins the study, we will expect him/her to be available for the whole period of the study. This is 3 months from the day he/she joins. Only those children who can complete the follow-up can take part in the study. Children who plan to move out of the area in the next 2 months cannot take part in the study.

The child must take all the medicine given by the study. The medicine we give is only for the child in the study. You must never share the medicine with your other children, even if they have signs of bilharzia.

Children who take part in the study can only take certain medicines. We therefore ask you not to buy extra medicines for your child from shops during the study. If your child is ill at any time during the 3 months of the study, you can contact the study doctor. For an emergency, you should bring your child to Kimbimbi sub-county hospital. If your child needs to stay in the hospital, we will pay for the treatment.

**What are the medicines that are being tested?**

In this study, we will compare children treated with Praziquantel or with Praziquantel combined with one of four common medicines used for treating malaria. The malaria medicines are Artesunate plus Sulfalene/pyrimethamine, Artesunate plus mefloquine or Artesunate plus amodiaquine or Duo-Cotexcin. All study drugs will be given as crushed tablets by mouth. We will compare five different treatment groups. One group will get only Praziquantel, which is the recommended treatment for bilharzia, and the other four groups will get a combination of Praziquantel and one of the four medicines for malaria. All these medicines are available in tablet form and will be taken only once per day.

**What are the alternatives for diagnosis or treatment?**

If you do not permit your child to take part in the study, your child will still receive the standard care for the treatment of bilharzia which is Praziquantel tablets.

**What are the side effects of any treatment received when taking part?**

All medicines we give may have side effects**.** There is a chance that your child will vomit after taking the medicines. If the child vomits within half an hour another treatment dose will be given. If the vomiting occurs after 30 minutes, no extra medicines will be given.

Praziquantel is well tolerated by most children. Some children can complain of headaches and may feel dizzy. Other children complain of coughing, feeling like vomiting, and may get tummy pain. In almost all children this is very mild. Malaria medicines are generally well tolerated by most children but may cause itching of the skin, stomachaches, and vomiting.

**What are the possible problems and risks of taking part?**

A small bruise may form, and the child may feel mild pain from the site on the finger where blood is taken. There is also a chance of infection when blood is drawn. This chance is very small because we always use clean materials. Your child may also be inconvenienced because the study will require your child to provide stool samples on more than one occasion and to return for scheduled follow-up visits.

**What are the possible benefits of taking part?**

*Treatment*

We hope that all treatments provided as part of this research will help your child. However, this cannot be guaranteed. The information we get from this study may help us to offer better treatment in the future to children who have intestinal bilharzia.

*Health care*

Your child will be tested for malaria and other intestinal worms, and if found, we will provide additional treatment for these. We will be responsible for the treatment of any illnesses that arise during the study—the study staff will arrange referral to an appropriate health facility and make payments for the medical care.

**What if new information becomes available?**

New information may become available about the medicines used in this study. If this happens the study doctor will tell you about it. If this happens, the study doctor will discuss with you whether you want your child to continue in the study. If you decide not to continue, the study will still arrange for your child’s care to continue. If you decide to continue in the study, you will be asked to sign an updated parental permission form.

**What happens when the research study stops?**

The medicines used in this study are available in the shops in Mwea or anywhere else.

**Will my child’s participation in this study be kept confidential?**

If you permit your child to participate in our research, we will keep his/her name and all the information that we get from you as part of this study private to the extent allowed by the law. The records may be reviewed by staff from KEMRI and the Kenyan Ministry of Health as part of their duty to oversee research. People from a safety board may also look at the records to check that the study is being carried out correctly. Any information about your child, that leaves the study site, will have the name and address removed so that your child cannot be recognized from it.

**What will happen to the results of the research study?**

We hope the results of this study will improve the way that children with bilharzia are treated. We will share the results of this study with many people so that other people will benefit from our results. You will be informed of the study results through a village meeting. We will also inform the staff of the Kenyan Ministry of Health, the Kimbimbi and Kerugoya Hospitals, and the company that makes the medicines we use. We will also present the results at conferences and publish them in a magazine of science. The name of your child will not be used in any report resulting from this study.

**Contact for further information.**

Should any further questions arise, or if you want to quit the study, or you think your child has been injured because of this study, please contact Prof Charles Obonyo [Tel 0724993118].

If you have any questions about your rights as a study participant, or if you want to talk about the study with someone, please contact the Scientific and Ethics Review Unit (SERU) at KEMRI headquarters in Nairobi, through [Tel 0717719477] or through Email: [seru@kemri.org](mailto:seru@kemri.org).

If you think your child has a medical problem that needs attention, please bring him/her to the study clinic located at Mwea sub-County Hospital.

We hope the results of this study will help to improve the treatment for intestinal bilharzia in this area. Thank you very much for your time.

IF THERE IS ANY PORTION OF THIS CONSENT EXPLANATION SHEET THAT YOU DO NOT UNDERSTAND, ASK THE INVESTIGATOR BEFORE SIGNING.

I acknowledge receipt of this Informed Consent Explanation.

Child's name _____________________________

Parent's/guardian's signature:________________________ Date____________

Parent's/guardian's Printed Name*:____________________________*

Witness's Signature: _________________________ Date: ___________

*Witness's Printed Name:_________________________*

# APPENDIX 3: INFORMED CONSENT AGREEMENT/CERTIFICATE

I, _____________________________________, having full capacity to consent for my child

(PARENT/GUARDIAN'S NAME)

_______________________, do hereby consent to his/her participation in the research study

(CHILD'S NAME)

entitled “**Efficacy and safety of Praziquantel alone or combined with Artesunate-based combinations in the treatment of children with *Schistosoma mansoni* in Mwea, Kirinyaga County**", under the direction of Prof Charles O. Obonyo. The implications of my child’s voluntary participation, the nature, duration, and purpose; methods and means by which it is to be conducted; and the inconveniences and hazards which may reasonably be expected have been explained to me by ______________________, and are outlined in the Informed Consent Explanation, which I have signed.

I have been allowed to ask questions concerning this experimental study, and my questions have been answered to my complete satisfaction. Should any further questions arise, I may contact Prof Charles Obonyo (of Kenya Medical Research Institute, Kisumu) at 0724993118 or Prof Sammy Njenga (of Kenya Medical Research Institute, Nairobi) at 0722945243.

I have been told that I may at any time during this study revoke my consent and withdraw my child from the study without prejudice; however, I may be requested to have my child undergo further examinations if, in the opinion of the doctor, such examinations are necessary for his/her wellbeing.

Child's name:__________________________________________

Parent's signature:____________________________ Date____________

Parent’s Printed Name*:____________________________*

**If illiterate,**

A literate witness preferably selected by the child’s parent, will sit in and witness the whole informed consent procedure and sign below:

I have witnessed the accurate reading of the consent form to the child’s parent, and the individual has had the opportunity to ask questions. I confirm that the individual has given consent freely.

**Witness's Printed Name**:_____________________ AND Thumb print of the child’s parent

**Witness's Signature**: _________________________ Date: ___________

I have accurately read or witnessed the accurate reading of the consent form to the parent or guardian of the potential participant and the individual has had the opportunity to ask questions. I confirm that the individual has given consent freely.

**Researcher’s Printed Name***:____________________________*

**Researcher’s Signature**:____________________________ Date____________

A copy of this Informed consent form has been provided to the parent or guardian of the participant. …………. (Initiated by researcher/assistant)

APPENDIX 4: TREATMENT DOSING TABLE

KIRINYAGA SCHISTO STUDY

TREATMENT SHEET

| Pupil’s Name |  | | | |
| --- | --- | --- | --- | --- |
| Age (years) |  | Class | |  |
| Body weight on Day 0 |  | |  | |

***Tick the Child’s treatment Assignment according to the Randomisation List:***

1= PZQ alone;

2= PZQ+AS+SMP;

3=PZQ +AS+MQ;

4=PZQ +DHAP’

5=PZQ +AS+AQ

| **TREATMENT DOSING TABLE (Circle the no of tablets according to the weight range)** | | | | | |
| --- | --- | --- | --- | --- | --- |
| Body weight (Kg) | Praziquantel (PZQ) | Coarinate (As+SMP) | Artequin (As+MQ) | Duocotexin  D-ARTEPP (DHAP) | Artesunate plus Amodiaquine  ASAQ (As+AQ) |
| 15.0-19.9 | 1 | ½ | 1 | 1 | 0.5 |
| 20.0-24.9 | 1.5 | 1 | 1 | 1.5 | 1 |
| 25.0-29.9 | 1.5 | 1 | 1 | 2 | 1 |
| 30.0-34.9 | 1.5 | 1 | 2 | 2 | 1 |
| 35.0-44.9 | 2 | 2 | 2 | 3 | 2 |
| 45.0-50.0 | 3 | 3 | 2 | 3 | 2 |
